# Supplementary material for: Determination of Cultivation Regions and Quality Parameters of Poria cocos by Near-Infrared Spectroscopy and Chemometrics
Source: Foods. 2022 Mar 21;11(6):892. doi: 10.3390/foods11060892 (PMC8956048; doi:10.3390/foods11060892)
Supplement: Supplementary file 1 [file foods-11-00892-s001.zip › foods-1627498-supplementary.pdf]

foods

MDPI

Supplementary Materials

Determination of Cultivation Regions and Quality Parameters of *Poria cocos* by Near-Infrared Spectroscopy and Chemometrics

Jing Xie [1], Jian-hua Huang [1], Guang-xi Ren [2], Jin Jian [1], Lin Chen [1], Can Zhong [1], Yuan Cai [1], Hao Liu [1], Rong-rong Zhou [1], Yu-hui Qin [1] and Shui-han Zhang [1,*]

[1] Hunan Academy of Chinese Medicine, Hunan University of Chinese Medicine, Changsha 410013, China; axxj2057@163.com (J.X.); jhhuang85@163.com (J.-h.H.); jinjian2016@163.com (J.J.); chenlin5202@126.com(L.C.); canzhong651@163.com (C.Z.); tcmyuanyuan@163.com (Y.C.); zrziqing@126.com (H.L.); rz172@georgetown.edu (R.-r.Z.); hntcmaqyh@126.com (Y.-h.Q.)

[2] College of Chinese Pharmacy, Beijing University of Chinese Medicine, Beijing 102488, China; renguangxiabc@163.com

* Correspondence: zhangshuihan@hnucm.edu.cn; Tel.: +86-0731-88881651

Table S1. *Poria cocos* samples information.

N0.[a]	Cultivation region[b]	Dataset	N0.	Cultivation region	Dataset
S.1	YN	Calibration	S.70	YN	Calibration
S.2	YN	Calibration	S.71	YN	Calibration
S.3	XQ	Calibration	S.72	DBM	Calibration
S.4	DBM	Calibration	S.73	DBM	Validation
S.5	DBM	Calibration	S.74	DBM	Calibration
S.6	YN	Calibration	S.75	DBM	Calibration
S.7	YN	Calibration	S.76	DBM	Validation
S.8	YN	Calibration	S.77	DBM	Calibration
S.9	YN	Calibration	S.78	XQ	Ignore
S.10	YN	Calibration	S.79	YN	Validation
S.11	YN	Calibration	S.80	DBM	Validation
S.12	YN	Calibration	S.81	XQ	Calibration
S.13	YN	Ignore	S.82	YN	Calibration
S.14	YN	Calibration	S.83	XQ	Calibration
S.15	YN	Calibration	S.84	DBM	Calibration
S.16	YN	Validation	S.85	XQ	Calibration
S.17	YN	Calibration	S.86	XQ	Calibration
S.18	YN	Calibration	S.87	XQ	Calibration
S.19	YN	Validation	S.88	XQ	Calibration
S.20	DBM	Calibration	S.89	XQ	Calibration
S.21	YN	Calibration	S.90	XQ	Calibration
S.22	YN	Validation	S.91	XQ	Calibration
S.23	YN	Calibration	S.92	XQ	Validation
S.24	YN	Calibration	S.93	DBM	Validation

S.25	YN	Validation	S.94	YN	Calibration
S.26	YN	Calibration	S.95	DBM	Calibration
S.27	YN	Calibration	S.96	DBM	Calibration
S.28	XQ	Calibration	S.97	DBM	Calibration
S.29	YN	Calibration	S.98	XQ	Calibration
S.30	YN	Calibration	S.99	XQ	Calibration
S.31	XQ	Calibration	S.100	DBM	Validation
S.32	YN	Calibration	S.101	DBM	Validation
S.33	YN	Calibration	S.102	DBM	Validation
S.34	YN	Calibration	S.103	YN	Calibration
S.35	YN	Validation	S.104	XQ	Calibration
S.36	YN	Calibration	S.105	DBM	Calibration
S.37	DBM	Calibration	S.106	DBM	Calibration
S.38	XQ	Calibration	S.107	XQ	Calibration
S.39	YN	Calibration	S.108	XQ	Calibration
S.40	XQ	Calibration	S.109	DBM	Validation
S.41	XQ	Calibration	S.110	DBM	Calibration
S.42	YN	Calibration	S.111	DBM	Calibration
S.43	YN	Calibration	S.112	DBM	Validation
S.44	YN	Calibration	S.113	YN	Calibration
S.45	YN	Calibration	S.114	DBM	Calibration
S.46	YN	Calibration	S.115	YN	Calibration
S.47	XQ	Calibration	S.116	DBM	Calibration
S.48	YN	Calibration	S.117	XQ	Calibration
S.49	YN	Calibration	S.118	DBM	Calibration
S.50	YN	Validation	S.119	YN	Calibration
S.51	DBM	Calibration	S.120	XQ	Calibration
S.52	DBM	Calibration	S.121	YN	Validation
S.53	DBM	Calibration	S.122	DBM	Calibration
S.54	DBM	Calibration	S.123	YN	Calibration
S.55	YN	Calibration	S.124	XQ	Calibration
S.56	DBM	Calibration	S.125	YN	Calibration
S.57	DBM	Validation	S.126	DBM	Calibration
S.58	DBM	Calibration	S.127	YN	Calibration
S.59	YN	Calibration	S.128	DBM	Validation
S.60	DBM	Calibration	S.129	YN	Calibration
S.61	DBM	Calibration	S.130	YN	Calibration
S.62	DBM	Calibration	S.131	DBM	Calibration
S.63	DBM	Calibration	S.132	DBM	Calibration
S.64	DBM	Validation	S.133	YN	Validation
S.65	DBM	Validation	S.134	YN	Calibration
S.66	DBM	Calibration	S.135	DBM	Calibration

S.67	DBM	Validation	S.136	XQ	Validation
S.68	DBM	Validation	S.137	DBM	Ignore
S.69	DBM	Validation	S.138	XQ	Calibration

[a], Sample number; [b], Sample from Cultivation region; YN, Yunnan region; XQ, Xiangqian region; DBM, Dabie Mountains region.
